# Clonal Evolution of High-Risk Chronic Lymphocytic Leukemia: A Contemporary Perspective

**DOI:** 10.3389/fonc.2021.790004

**Published:** 2021-12-16

**Authors:** Marwan Kwok, Catherine J. Wu

**Affiliations:** ^1^ Institute of Cancer and Genomic Sciences, University of Birmingham, Birmingham, United Kingdom; ^2^ Centre for Clinical Haematology, Queen Elizabeth Hospital Birmingham, Birmingham, United Kingdom; ^3^ Department of Medical Oncology, Dana-Farber Cancer Institute, Boston, MA, United States; ^4^ Harvard Medical School, Boston, MA, United States; ^5^ Broad Institute of MIT and Harvard, Cambridge, MA, United States; ^6^ Department of Medicine, Brigham and Women’s Hospital, Boston, MA, United States

**Keywords:** chronic lymphocytic leukemia, clonal evolution, intratumoral heterogeneity, single-cell analysis, Richter syndrome

## Abstract

Clonal evolution represents the natural process through which cancer cells continuously search for phenotypic advantages that enable them to develop and expand within microenvironmental constraints. In chronic lymphocytic leukemia (CLL), clonal evolution underpins leukemic progression and therapeutic resistance, with differences in clonal evolutionary dynamics accounting for its characteristically diverse clinical course. The past few years have witnessed profound changes in our understanding of CLL clonal evolution, facilitated by a maturing definition of high-risk CLL and an increasing sophistication of next-generation sequencing technology. In this review, we offer a modern perspective on clonal evolution of high-risk CLL, highlighting recent discoveries, paradigm shifts and unresolved questions. We appraise recent advances in our understanding of the molecular basis of CLL clonal evolution, focusing on the genetic and non-genetic sources of intratumoral heterogeneity, as well as tumor-immune dynamics. We review the technological innovations, particularly in single-cell technology, which have fostered these advances and represent essential tools for future discoveries. In addition, we discuss clonal evolution within several contexts of particular relevance to contemporary clinical practice, including the settings of therapeutic resistance to CLL targeted therapy and immunotherapy, as well as Richter transformation of CLL to high-grade lymphoma.

## Introduction

Clonal heterogeneity and evolution are among the most fundamental properties of cancer. Through a reiterative process of clonal proliferation, diversification and Darwinian selection, cancers continually adapt within the host microenvironment, progressively acquiring and accumulating enabling attributes that allow them to develop and expand (1). Intratumoral heterogeneity fuels this evolutionary process by providing a diverse pool of candidates from which the fittest parental tumor subclone is selected and propagated to the subsequent generation. Intratumoral heterogeneity is underpinned by genetic heterogeneity with different tumor subclones each harboring a unique constellation of genetic aberrations. In addition to genetic diversity, intratumoral heterogeneity also manifests in other dimensions. These include variation in transcriptional cell states, epigenetic programs, and tumor-immune interactions among different cancer cell populations within the tumor ecosystem (2, 3). Studying clonal evolution thus allows the capture of this dynamic, iterative process that results in tumor initiation and progression, and that dictates subsequent treatment response and relapse.

Chronic lymphocytic leukemia (CLL), a malignancy of CD19^+^ CD5^+^ B lymphocytes, offers an informative disease model to study cancer evolution. First, CLL is characterized by clinical heterogeneity that encompasses a range of disease trajectories including rapid progression, treatment refractoriness and high-grade transformation at one end of the spectrum (4, 5), to a highly stable clinical course or even spontaneous disease regression at the opposite end (6, 7). This allows tumor evolution to be studied across a range of differing clinical contexts. Second, the typically protracted disease course in CLL allows clonal evolution to be deciphered through frequent longitudinal sampling over a period of many years, thereby providing a wealth of data on evolutionary dynamics at high temporal resolution. Third, CLL cells circulate continuously between peripheral blood and the lymph node and bone marrow compartments (8, 9). Tumor samples of high purity and quantity can thus be readily obtained from peripheral blood. In addition, lymph node and bone marrow specimens can also be accessed with relative ease complementing peripheral blood samples to allow the comprehensive study of tumor co-evolution with the immune microenvironment (10).

The clinical heterogeneity of CLL necessitates identification of biological correlates of high-risk CLL, in order to define the patient population most at risk of CLL progression that merits close monitoring and focused study. Over the past two decades, biomarkers of high-risk CLL have evolved with our increasingly sophisticated understanding of CLL biology. Concurrently, advances in bulk sequencing and more recently integrative single-cell sequencing technology have facilitated the longitudinal study of CLL clonal evolution in this patient group. These studies have illuminated our understanding of CLL clonal architecture and the complex clonal evolutionary dynamics that give rise to CLL progression, resistance to different CLL treatments and high-grade transformation, revealing diverse biological processes and novel mechanisms. In this review, we will appraise recent advances in our understanding of intratumoral heterogeneity and clonal evolution in patients with high-risk CLL, and discuss the technological innovations that have facilitated this understanding. We will focus particularly on clonal evolution within several topical contexts, including the settings of therapeutic resistance to CLL targeted therapy and immunotherapy, as well as Richter transformation of CLL to high-grade lymphoma.

## The Evolving Definition of High-Risk CLL

**Figure 1:**
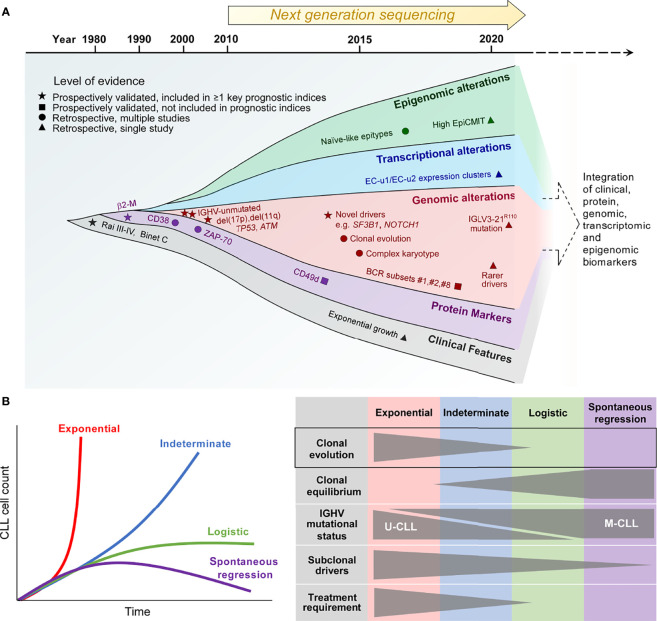
The biological traits of high-risk CLL. **(A)** The definition of high-risk CLL evolving from the traditional reliance on a single or several discrete biomarkers towards the multimodal integration of multiple biomarkers that reflect the clinical, phenotypic, genetic, transcriptional and epigenetic properties of high-risk CLL. **(B)** Patterns of CLL growth dynamics, highlighting the importance of clonal evolution for exponential growth and CLL progression. β2-M, β2-microglobulin; IGHV, immunoglobulin heavy chain variable region; IGLV, immunoglobulin light chain variable region; EpiCMIT, epigenetically-determined cumulative mitoses; U-CLL, IGHV unmutated CLL; M-CLL, IGHV mutated CLL.

Our understanding of what constitutes high-risk CLL has evolved considerably over recent years (**Figure 1A**). Conventional definitions of high-risk CLL are based on clinical information supplemented by a limited number of adverse genetic and flow cytometry-based biomarkers. Novel definitions of high-risk CLL, on the other hand, reflect the integration of a multitude of biological information pertaining to the tumor that serves to enhance prognostic stratification. We discuss herein the various definitions of high-risk CLL which provide an important basis for the study of CLL clonal evolution.

### Conventional Definitions of High-Risk CLL

Historically, high-risk CLL was defined solely on the basis of clinical features, with the presence of cytopenias being surrogates of CLL risk, as reflected in the Rai and Binet staging systems (11, 12). With the ubiquitous use of flow cytometry for CLL diagnosis, subsequent developments have linked several CLL cell-surface proteins, such as a high level of CD38, ZAP-70 and/or CD49d expression, to adverse prognosis (13–15). At the same time, our increasing appreciation of the role of B-cell receptor (BCR) signaling as a major driver of CLL proliferation has led to the identification of two distinct biological subtypes of CLL distinguished by the status of somatic hypermutation within the immunoglobulin heavy chain variable region (IGHV). IGHV-unmutated CLL (U-CLL) is associated with a higher progression risk compared to its IGHV*-*mutated counterpart (M-CLL) (13, 16), relating in part to higher capacity for BCR signaling in the former (17, 18). BCR stereotypy is a feature of CLL, and specific stereotyped subsets, such as subset #2 characterized by IGHV3-21/IGLV3-21 gene usage, as well as subsets #1 and #8, confer increased disease aggressiveness (19–22). Notably, subset #2 is linked to an aggressive CLL clinical course independent of IGHV mutational status (19). Recent work has shown that subset #2 CLL uniformly harbors the IGLV3-21^R110^ mutation (23). Moreover, non-stereotyped CLL possessing this mutation exhibits similar adverse biological and clinical characteristics to stereotyped subset #2 CLL, suggesting that this subset could be defined by the IGLV3-21^R110^ mutation.

In addition to IGHV and cell-surface biomarkers, fluorescence *in-situ* hybridization (FISH) has enabled CLL risk stratification into distinct cytogenetic risk categories with del(17p) carrying the highest risk, del(11q) and trisomy 12/normal FISH conferring high and intermediate risk respectively, and isolated del(13q14) being associated with lower risk (24). Moreover, early studies into CLL molecular genetics have established the adverse prognostic impact of somatic mutations involving *TP53* and *ATM* (25–27). These prospectively validated, conventional biomarkers of high-risk CLL, particularly del(17p) as well as IGHV and *TP53* mutational status, continue to find relevance in contemporary clinical practice, and are featured within widely used prognostic indices such as the CLL International Prognostic Index (CLL-IPI) (28). Finally, the importance of complex karyotype identified by chromosome banding analysis and/or genomic microarrays has recently arisen, with complex karyotype conferring inferior outcome independently of the CLL-IPI (29–31).

### Re-Defining High-Risk CLL Through Multimodal Integration of Biological Traits

The advent of next-generation sequencing technology in the past decade heralded an expansion in our knowledge of the CLL genome, epigenome and transcriptome (32, 33). In line with this, additional biomarkers of high-risk CLL have emerged. First, bulk whole exome sequencing (WES) and whole genome sequencing (WGS) efforts in large patient cohorts have provided comprehensive atlases of recurring CLL genomic alterations with putative functional significance encompassing both single-nucleotide variations (SNVs) and copy-number alterations (CNAs), revealing hitherto unknown genomic CLL drivers (34, 35). Some of these novel drivers, such as *SF3B1* and *NOTCH1* mutations, identified patients at higher risk of disease progression as well as disease recurrence after chemotherapy-based CLL therapy (34, 36, 37). Second, genome-wide methylation studies have identified three CLL epigenetic subtypes differentiated on the basis of their methylation profiles (38–40). These distinct epitypes reflect the developmental maturation state of the putative normal B-cell counterpart from which the different CLL subtypes are derived. Of these epitypes, naïve-like CLL, which is less epigenetically mature than the other epitypes (i.e. intermediate and memory-like CLL) and possesses ability for further epigenetic programming, is associated with higher progression risk. Furthermore, within individual epitypes, a higher epigenetically-determined cumulative mitoses (epiCMIT) score, which reflects more extensive CLL proliferation history, correlates with adverse prognosis (41).

A recent large-scale analysis of the CLL transcriptome has yielded 8 gene expression clusters (ECs) with prognostic significance, each corresponding to a distinct transcriptional profile that reflects a unique CLL phenotypic state (42). On this basis, U-CLL clusters into two subtypes (EC-u1, EC-u2), whereas M-CLL can be clustered into four subtypes (EC-m1, EC-m2, EC-m3 and EC-m4). The remaining two clusters are EC-i and EC-o respectively, the former closely associates with the intermediate CLL epitype, while the latter does not correlate with any previously defined CLL group. The two EC-u clusters confer adverse risk, with EC-u1 and EC-u2 having similarly short progression-free and overall survival.

Some of these newer biological correlates of high-risk CLL, such as adverse epitypes and ECs as well as high epiCMIT, require further prospective validation. Nevertheless, their characterization has offered opportunity for the integration of genetic, transcriptional, epigenetic, phenotypic and clinical parameters to refine prognostic stratification, thereby providing a more accurate definition of high-risk CLL. Indeed, a recent multicenter effort by Knisbacher and colleagues utilizing data acquired from hundreds of patient samples have generated multivariate prognostic models that incorporate these different parameters (42). While much of our current understanding of CLL clonal evolution is derived from studies based on conventional definitions of high-risk CLL, as well as other adverse clinical features such as therapeutic resistance and high-grade transformation, contemporary prognostic classification constructed upon the basis of biomarker integration provides a useful foundation for the future study of evolutionary dynamics in high-risk CLL.

### Clonal Evolution as a Determinant of High-Risk CLL

The clinical heterogeneity of CLL is quite evident in highly disparate clinical trajectories that can be observed amongst patients. An indolent or slowly progressing clinical course is observed in the majority of CLL patients, with rapid disease progression and spontaneous regression at the two extremes. These different clinical trajectories are mirrored by differences in clonal growth dynamics (43). Growth patterns that have been demonstrated in CLL include exponential unbounded growth, as well as logistic growth that stabilizes at a specific carrying capacity and plateaus over time, with exponential growth being considered higher risk as evidenced by a shorter time to treatment compared to logistic growth (**Figure 1B**, left panel). In addition, an indeterminate category falls between these two clearly defined growth patterns.

Through the analysis of serial samples from CLL patients, Gruber and colleagues linked differences in CLL growth trajectories to variations in clonal genetic composition as well as the extent of clonal evolution (43). Compared to patients exhibiting logistic CLL growth, patients who exhibit exponential growth are more likely to have U-CLL, harbor greater number of CLL driver mutations, and display more extensive clonal evolution marked by more profound shifts in subclonal proportions over time. In contrast, clonal equilibrium, wherein subclonal proportions remain stable over time, is more commonly observed in patients with logistic growth (**Figure 1B**, right panel). Clonal equilibrium associated with a relative paucity of subclonal genomic drivers also appears to be the norm among the rare cases of spontaneously regressing M-CLL, as reported recently by Kwok et al. (7). These findings corroborate earlier work that has established a correlation between CLL clonal evolution and adverse prognosis (34, 44). Together, these studies highlight the role of clonal evolution in shaping the natural history of individuals with high-risk CLL.

## Harnessing Technological Advances to Interrogate CLL Clonal Evolution

**Figure 2:**
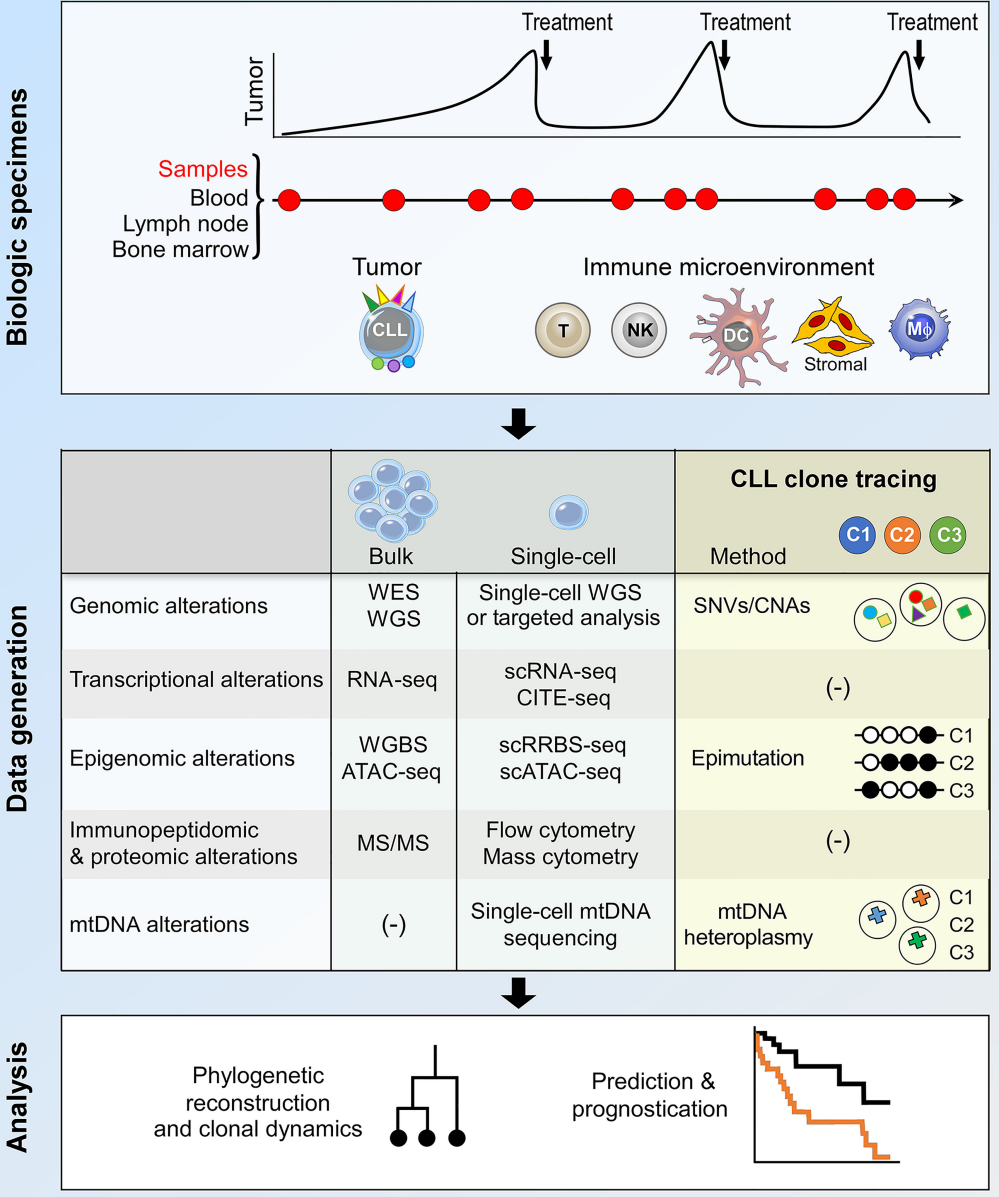
Integrative bulk and single-cell analysis of CLL clonal dynamics. The current approach to the study of CLL clonal evolution is summarized, highlighting the use of biological specimens (top panel) and methodologies for data generation and analysis (middle and bottom panels respectively). Longitudinal samples collected at various time points during the clinical course of a patient allow analysis of both the tumor and the immune microenvironment. Various bulk and single-cell data generation approaches can be used to interrogate biological alterations that underpin clonal evolution. Within the context of single-cell analysis, lineage tracing techniques facilitate the identification of CLL subclones and the integration of multimodal data pertaining to individual subclones. The data thus generated can be used for phylogenetic reconstruction, analysis of subclonal dynamics and clinical prognostication. DC, dendritic cell; Mφ, macrophage; WES, whole exome sequencing; WGS, whole genome sequencing; SNV, single-nucleotide variation; CNA, copy number alteration; mtDNA, mitochondrial DNA; scRNA-seq, single-cell RNA-seq; CITE-seq, cellular indexing of transcriptomes and epitopes by sequencing; WGBS, whole genome bisulfite sequencing; scRRBS-seq, single-cell reduced representation bisulfite sequencing; scATAC-seq, single-cell sequencing assay for transposase-accessible chromatin; MS/MS, tandem mass spectrometry.

The study of clonal evolution in cancer is reliant on data generation and analytical platforms that are capable of delineating clonal architecture and subclonal phylogenetic relationships from longitudinal patient samples (3, 45). In CLL, these are usually peripheral blood samples, occasionally complemented by bone marrow and lymph node specimens to allow the study of CLL tumor-immune co-evolution within important microenvironmental niches (**Figure 2**, upper panel). Bulk sequencing analysis of these samples have facilitated much of our current understanding of CLL clonal evolution, including in high-risk patients. On the other hand, recent advances in single-cell sequencing technology provide opportunities for the interrogation of CLL clonal evolution at unprecedented resolution, which will likely transform our understanding of evolutionary mechanisms in high-risk CLL (**Figure 2**, middle and lower panel). These two approaches will be discussed in turn.

### Bulk Analysis of CLL Clonal Evolution

Computational analysis of bulk WES and WGS data allows identification of genomic drivers that are more recurrent than expected by chance, and are inferred to increase clonal fitness and drive oncogenesis, distinguishing them from passenger somatic alterations that are co-incidental and do not confer growth advantage or directly drive initiation or progression. Moreover, the computational integration of read depth and variant allelic frequencies of somatic mutations permits estimation of the cancer cell fraction (CCF) of each driver that corrects for CLL sample purity and chromosomal copy number alteration (34, 44). Analysis of longitudinal samples from individual patients using bulk WES or WGS thus permits inference of CLL clonal architecture and subclonal phylogenetic relationships from the coordinated patterns of temporal fluctuations in CCF. CLL genomic drivers that are consistently clonal (i.e. present at CCF >0.95), such as *MYD88* mutation, trisomy 12 and del(13q14), reflect early genomic events that mediate CLL development. On the other hand, other driver mutations that are typically present in only a fraction of CLL cells are subclonal and likely represent later events that arise from subclonal selection. Subclonal mutations are thought to confer enhanced clonal fitness and drive CLL progression (34, 44). Examples of subclonal CLL drivers include mutations within *ATM*, *SF3B1*, *TP53* and *BIRC3.* In addition to genomic analyses, transcriptome profiling studies (e.g. bulk RNA-seq) demonstrate global transcriptional changes that often accompany genetic clonal evolution (46–48), while epigenomic studies such as genome-scale DNA methylation analyses on bulk CLL cell populations reveal remarkable intratumoral epigenetic heterogeneity that fuels clonal evolution (49).

Despite their proven utility, a fundamental limitation of clonal evolution studies based on bulk sequencing methodologies lies in their inability to resolve with precision subclonal phylogenetic relationships at low CCFs, because the capacity to detect rare subclonal genomic events is often limited by sequencing read depth. Moreover, bulk analyses do not readily permit an integrative analysis of the genetic, epigenetic and transcriptional dynamics of individual CLL subclones that is essential to understand complex evolutionary mechanisms. These limitations can be addressed through contemporary approaches that leverage multidimensional single-cell sequencing technology.

### Single-Cell Analysis of CLL Clonal Evolution

Single-cell analysis, by definition, allows high-resolution reconstruction of clonal phylogenetic architecture, as well as the determination of cell state dynamics in relation to genetic lineage history, through the integration of multiple strata of biological information across longitudinal time points at single-cell resolution (3, 45). The latter is achieved through coupling single-cell RNA-seq (scRNA-seq) with single-cell reduced representation bisulfite sequencing (scRRBS-seq), single-cell chromatin accessibility assays [e.g. single-cell sequencing assay for transposase-accessible chromatin (scATAC-seq)] and/or single-cell WGS or genotyping, performed simultaneously on RNA and DNA extracted from the same cells, thereby linking cellular transcriptional states with gene regulatory networks and genomic aberrations. The deconvolution of CLL subclonal dynamics through an integration of multimodal single-cell biological data necessitates the deployment of robust methodologies to track individual subclones over time, a process known as lineage tracing. The use of synthetic sequencing barcodes enables prospective lineage tracing within *in vitro* and *in vivo* CLL models (50), and is being increasingly explored across cancer systems. With the currently available tools, however, such a strategy is largely unfeasible to use in primary biospecimens from CLL patients given the well-known obstacles to efficiently introduce such barcodes into primary B cells. Instead, retrospective approaches for lineage tracing that exploit heritable native barcodes such as SNVs/CNAs, mitochondrial DNA (mtDNA) heteroplasmy or epimutations have been used to identify and mark each individual subclone (50–57). These approaches will each be elaborated in more detail.

### Novel Approaches for Lineage Tracing in CLL at Single Cell Resolution

An established method for lineage tracing involves the tracking of SNVs and/or CNAs that are present within individual subclones. Such an approach utilizes experimental platforms that integrate the sequencing of single-cell genomes and transcriptomes (e.g. G&T-seq, sci-L3-RNA/DNA) (51, 52), and others that incorporate single-cell somatic genotyping within scRNA-seq [e.g. targeted RNA qPCR, Genotyping of Transcriptomes (GoT)] (53, 54). However, a major limitation of this approach is the fact that SNVs and CNAs can be infrequent in certain CLL subclones. They are also vulnerable to selection pressure during the course of clonal evolution, therefore lacking the required stability and consistency for a lineage marker. Moreover, single-cell WGS has limited scalability with allelic dropout issues, while GoT is challenging to use in the context of lowly expressed genes. Alternative lineage markers, such as mtDNA heteroplasmy and epimutations, provide opportunities to overcome these barriers.

Mitochondrial DNA heteroplasmy are naturally occurring, stochastic mtDNA mutations that can serve to identify individual tumor subclones (55). Such mutations are particularly attractive for lineage tracing owing to their consistent and stable propagation within a specific subclonal lineage from one generation to the next. In a proof-of-concept study, Penter and colleagues applied mitochondrial scATAC-seq (mtscATAC-seq), which provides conjoint readout of mtDNA mutations and chromatin accessibility information, to the analysis of clonal dynamics in patients with high-risk CLL (56). This study confirms the ability of distinct mtDNA mutations to stably mark separate CLL subclones with different chromatin states. Moreover, the use of mtDNA mutations as lineage markers allow efficient tracking of the varied temporal dynamics of CLL subclones in response to different treatment modalities and during Richter transformation. Notwithstanding uncertainties surrounding the role of mtDNA heteroplasmy in CLL pathogenesis, and our as yet nascent understanding of mtDNA dynamics, mtDNA-based lineage tracing represents a major technical advance for the single-cell analysis of CLL clonal evolution.

Similar to mtDNA mutations, stochastic DNA methylation changes known as epimutations are heritable marks that can be adopted for lineage tracing (57). Epimutations that lead to the random site-specific gain or loss of DNA methylation are accumulated during DNA replication and cell division, reflect cellular proliferation history, and can serve as epigenetic molecular clocks. Landau and colleagues showed that epimutations are ubiquitous features of CLL cells, readily detectable by RRBS-seq across large swathes of the CLL genome (49). Applying scRRBS-seq and scRNA-seq to longitudinal CLL samples, Gaiti et al. demonstrated the capability of epimutation information to identify individual CLL subclones with distinct genetic and/or transcriptional profiles, thereby enable accurate reconstruction of clonal phylogenies and characterization of CLL subclones with differential treatment response (57).

Finally, recent innovations in synthetic barcode technology for single-cell sequencing promise to revolutionize the use of *in vitro* and *in vivo* models to interrogate CLL clonal evolution. An example of this is ClonMapper that enforces expression of unique single-guide RNA (sgRNA) barcodes within single cells (50). These barcodes can be captured subsequently during scRNA-seq, thereby coupling clonal identity with single-cell transcriptomics, and allowing for the isolation of subclones of interest for further integrative multiomics study. To illustrate the applicability of this technology for modelling clonal evolution in high-risk CLL, Gutierrez and colleagues implemented this platform to monitor subclonal diversification in a CLL cell line in response to treatment, uncovering a host of genomic and transcriptomic cell state changes, and unique subclonal dynamics (50). Although this study was carried out *in vitro*, one can envisage the use of similar barcode technology within various murine models of CLL, including Eµ-TCL1, CLL patient-derived xenografts and newer CRISPR/Cas9-engineered transgenic models (58–61), opening up unprecedented opportunities for the prospective investigation of clonal evolution under experimental CLL therapies or therapeutic combinations.

### Analysis of Tumor-Immune Co-Evolution

Tumor cells reside within microenvironmental niches where they constantly interact with immune cells. These tumor-immune interactions contribute to shaping clonal evolution, and our appreciation of their importance have led to a growing impetus for the study of tumor-immune dynamics which is dependent upon both intrinsic tumor immunogenicity and the extrinsic immune microenvironment. With regard to tumor immunogenicity which in turn is determined by its antigenic landscape, mass spectrometric analysis of CLL major histocompatibility complex (MHC) class I and II immunopeptidomes, complemented by computational analysis of genomic and transcriptomic data (62, 63), enables characterization of the CLL antigenic landscape and its evolution over time. In relation to the extrinsic immune microenvironment, single-cell transcriptomics and epigenomics, applied across longitudinal patient samples or within *in vivo* models, enable accurate delineation of different immune populations as well as the characterization of dynamic immune cell states and tumor-immune interactions.

Altogether, these exciting new technological innovations will undoubtedly further advance our understanding of clonal evolution in high-risk CLL. In order to appreciate the context upon which future discoveries can be made, our current understanding of the molecular basis of intratumoral heterogeneity and clonal evolution in CLL is reviewed in the next sections.

## The Molecular Basis of CLL Intratumoral Heterogeneity and Evolution

**Figure 3:**
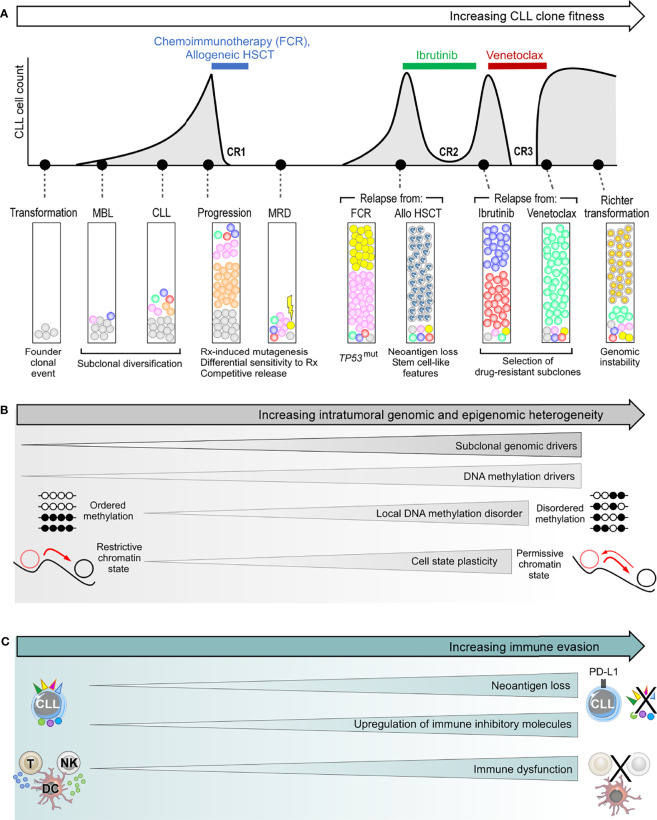
The genetic and non-genetic basis of intraclonal heterogeneity and clonal evolution in CLL. **(A)** Clonal evolution during the clinical course of a typical patient with high-risk CLL, illustrating subclonal dynamics during natural progression, as well as different patterns of subclonal selection that accompany treatment with chemoimmunotherapy, allogeneic HSCT (allo-HSCT), ibrutinib and venetoclax. **(B)** The various sources of intratumoral genetic and epigenetic heterogeneity that underpin clonal evolution in CLL. **(C)** The mechanisms of immune evasion that can facilitate clonal evolution. FCR, fludarabine, cyclophosphamide and rituximab combination; HSCT, hematopoietic stem cell transplantation; CR, complete response; MBL, monoclonal B-cell lymphocytosis; MRD, measurable residual disease.

Intratumoral heterogeneity in CLL is commonly understood to stem from genetic heterogeneity as a consequence of mutations and other genetic alterations. However, possession of genetic drivers is not a prerequisite for CLL progression. Indeed, sequencing efforts have failed to identify such drivers in some instances of CLL progression and relapse (43). Conversely, the presence of genetic drivers does not inevitably result in disease progression (64), as evidenced by patients harboring these drivers who remain at the monoclonal B-cell lymphocytosis (MBL) or indolent CLL stage for many years (65–67). Patients with CLL who spontaneously regress despite harboring *TP53* mutations in their CLL clone provide further testament to the notion that the mere presence of CLL genetic drivers is insufficient to drive clonal evolution or disease progression (7). Multiple lines of evidence now support an interplay between genetic, epigenetic and transcriptional cell states, as well as microenvironmental and immune factors in contributing to CLL intratumoral heterogeneity and clonal evolution (49, 57, 68–70). These various sources of CLL heterogeneity will be discussed below, highlighting their role in driving clonal evolution (**Figure 3**).

### The Genetic Basis of CLL Intratumoral Heterogeneity

Genetic variation underpins much of the clonal heterogeneity in CLL, with an average mutation rate of 0.6 to 1.1 per megabase but with a wide variation across individuals (range, 0.03 to 2.3) (34, 35, 42, 44, 71). In addition to SNVs and CNAs, more profound genomic disruptions such as kataegis (localized hypermutation hotspots), chromothripsis (localized clusters of hundreds to thousands of chromosomal rearrangements within a single or several chromosomes) and chromoplexy (complex chromosomal rearrangements involving multiple chromosomes) have been described in CLL (35, 71, 72). Analyses of CLL mutational signatures have revealed several mutagenic processes as likely contributors to its genetic heterogeneity. These include age-related mutagenesis reflecting a predilection of CLL for the elderly population, as well as activation-induced cytidine deaminase (AID) and APOBEC-related mutagenic processes that reflect CLL as a mature B cell malignancy putatively derived from antigen-experienced B cells with capacity for AID/APOBEC-mediated somatic hypermutation (42, 71, 73). Recent analyses of CLL WGS data have additionally identified mutational signatures arising from oxidative stress (42), as well as from DNA polymerase activity, replication slippage and defective DNA repair (74), the latter reflecting replication errors as sources of genomic alteration. Genomic instability in CLL may be further promoted by permissive genetic contexts resulting from loss of cell cycle control (e.g. *TP53* or *CDKN2A/B* mutation), DNA damage response (DDR; e.g. *ATM* or *SAMHD1* mutation) (75) or telomere maintenance (e.g. *POT1* mutation) (76), giving rise to additional genetic heterogeneity.

### Genetic Evolution in CLL Development and Natural Progression

Clonal evolution studies based on the analysis of WES or WGS data have enabled the characterization of a founder clone in CLL patients wherein somatic mutations are present (44, 64). These clonal mutations likely include initial leukemogenic drivers contributing to malignant transformation. On the other hand, subclonal mutations that subsequently emerge drive CLL clonal evolution. Underlining the importance of the latter, Landau et al. showed that many subclonal drivers expand towards clonality concomitantly with disease progression, and that the presence of such drivers confers adverse prognosis (34, 44). Murine models also support the role of specific mutations as drivers of CLL progression, either individually or in combination (61, 77–81).

The corresponding stages of normal B cell maturation during which founder mutations begin to emerge and subclonal genomic diversification commences is a matter of considerable contention. For patients with MBL who subsequently progress to CLL, several studies have demonstrated that the mutational burden as well as the clonotypic and genomic landscapes of MBL and CLL are largely similar (66, 67, 82, 83), indicating that the process of subclonal genomic diversification that drives leukemic progression is likely to have been established during or prior to the MBL stage. Provocatively, CLL driver mutations have also been reported within hematopoietic stem cells (HSCs) as well as myeloid and lymphoid progenitor cells in some patient with CLL, albeit at low variant allelic frequencies (84, 85), akin to age-related clonal hematopoiesis (55, 86–88). The potential role of HSCs in the pathogenesis of CLL has been corroborated by experiments which demonstrated the ability of HSCs from CLL patients to produce *de novo* CLL-like disease with distinct IGHV rearrangements upon xenotransplantation into immunodeficient mice (89). The occurrence of phylogenetically unrelated oligoclonal IGHV rearrangements in a substantial proportion of patients also supports the proposition that CLL-initiating events could predate somatic V(D)J recombination (90). On the other hand, clonally-related IGHV variants could reflect subclonal diversification events occurring downstream of the CLL founder clone, highlighting the importance of IGHV-D-J sequencing to track clonal evolution in CLL (91). If indeed the initiating genomic alteration in some patients occurs at the level of HSCs or progenitor cells, it is conceivable that further genomic events occur downstream of this, possibly after commitment to the B-cell lineage, and prior to establishment of the founder CLL clone.

The subsequent clinical course following CLL establishment is highly divergent and is underpinned by heterogeneous evolutionary dynamics. While clonal equilibrium features in the majority of patients within the treatment-naïve setting, clonal evolution is observed in others **(** **Figure 3**) (44, 64, 92). Gruber et al. showed that even in patients exhibiting clonal equilibrium that typically manifests in logistic growth, there is a varying degree of subclonal competition resulting in mild fluctuations in subclonal proportions, and such complex intraclonal dynamics can result in net carrying capacity (43). In some cases, certain subclones can acquire exponential growth which could be a harbinger for subsequent disease acceleration. Moreover, as recently suggested by *in vitro* models, the persistence of clonal equilibrium may also be predicated upon dynamic intracellular interactions between subclonal CLL populations, the disruption of which may perturb this delicate balance resulting in specific subclonal outgrowth (50).

Clonal evolution, on the other hand, can be considered from a purely genetic standpoint as the natural consequence of differential growth accelerations conferred by the different genetic drivers within competing subclones. In CLL, the strongest growth accelerations appear to be associated with tumor suppressor genes such as *TP53* or *ATM*, with other drivers such as *ASXL1*, *GNB1*, *XPO1*, and *KRAS* also conferring substantial growth acceleration (43). Differential growth rates conferred by different drivers are also evident from a CLL cell line model engineered to express different driver mutations (93). The heterogeneous growth rates of competing subclones result in linear or branching patterns of clonal evolution, both of which are frequently observed in CLL. Linear evolution is characterized by the sequential acquisition of advantageous alterations within a subclonal lineage that allows it to outcompete and dominate all antecedent subclones *via* successive selective sweeps. Branching evolution, on the other hand, is characterized by multiple subclones co-evolving in parallel, either mutating the same driver (‘convergent evolution’) or different drivers (‘divergent evolution’) to compete for dominance.

### Genetic Evolution in Response to CLL Treatment

CLL treatment has consistently been shown to fuel clonal evolution, resulting in fitter subclones with higher growth rates at the time of relapse. The resultant evolutionary pattern can be seen as a function of the pre-existing subclonal landscape at the time of treatment, and the nature of that CLL treatment. The importance of the former is evidenced from observations that a CLL harboring a wider spectrum of genetic alterations, more subclonal drivers, more complex subclonal landscapes, and exponential growth at the time of treatment is likely to experience more profound clonal shifts in response to treatment (43, 44). This can be understood from the availability of a wider pool of genetically fitter subclones from which therapeutic resistance could develop. Indeed, in many instances the dominant CLL subclone at the time of relapse is already pre-existing as a minor subclone at the time of treatment (34, 44, 64, 92), reflecting treatment-induced selection of resistant subclones. In many cases, these subclones with selective advantage are also those possessing higher levels of genomic instability that predispose them to additional genomic alterations, resulting in higher potential for further genetic diversification.

With regards to the nature of the CLL treatment, different treatment modalities exert differing selective pressures, the consequence being that subclones harboring distinct genetic drivers may have different strengths of fitness advantage under different treatments. For instance, *TP53*-mutant and/or del(17p) CLL subclones are almost invariably selected under chemotherapy-based treatments (34, 94, 95), whereas this is often not the case with targeted therapy (47, 96). In addition, chemotherapy-induced mutagenesis can contribute to genetic diversification, a process not seen with targeted therapy. Nevertheless, all CLL treatments regardless of modality create some manner of an evolutionary bottleneck (44, 97). This alters the subclonal landscape by removing incumbent subclones in favor of those with diminished sensitivity to treatment or enhanced survival and growth advantage that are ‘competitively released’ into the tumor ecosystem following treatment cessation.

### The Epigenetic Basis of CLL Cell State Heterogeneity

Notwithstanding the unequivocal impact of genomic aberrations, clonal fitness is not exclusively determined by genetic features. Indeed, there are documented examples of CLL subclones with exceptional growth rates but no identifiable genetic drivers (43). Moreover, CLL progression or relapse following treatment does not invariably coincide with the expansion of subclones harboring specific genetic alterations (64). These observations imply the existence of other factors influencing clonal fitness. In recent years, studies harnessing scRNA-seq technology have demonstrated substantial CLL transcriptional changes accompanying disease evolution, with transcriptional features evolving alongside genetic features (53, 98). Moreover, apparently divergent patterns of genetic evolution could potentially mask consistent transcriptional changes occurring in genetically distinct subclones, resulting in convergent evolution at the transcriptional level that could foster gene expression cohesiveness among CLL cells (53). Recent work has also revealed profound epigenetic cell state heterogeneity in CLL that influences clonal fitness and underpins clonal heterogeneity at the genetic, transcriptional and phenotypic levels (49, 57, 69).

Our current understanding of the epigenetic landscape in CLL is derived, to a large extent, from DNA methylation studies (38–40). These studies have shown that the CLL methylome is characterized by global gene body hypomethylation, accompanied by focal hypermethylation at promoters of tumor suppressor genes. The latter results in functional inactivation of these genes, thus representing a non-genetic mechanism through which CLL subclones could acquire fitness advantage. Tumor suppressor genes that are inactivated through this mechanism are known as DNA methylation drivers. A recent study by Pan and colleagues reported 122 putative DNA methylation drivers in CLL, of which 3 were functionally validated (70). These were *DUSP22*, *RPRM* and *SASH1*, which impact upon diverse leukemogenic processes including oncogenic signaling, cell cycle dysregulation and CLL migration. Analysis within patient cohorts showed that the presence of such drivers confers adverse prognosis. Moreover, longitudinal study revealed the emergence of novel DNA methylation drivers at the time of CLL relapse, thus substantiating their role as drivers of clonal evolution and disease progression.

DNA methylation studies also revealed that the inherited DNA methylome of a CLL represents an epigenetic imprint of both its putative cell of origin and proliferation history (38–41). In most patients, global patterns of CLL DNA methylation exhibit longitudinal stability (99), with evolution from the ancestral methylome being reported only in some individuals during CLL progression accompanying genetic evolution (68). Nevertheless, CLL cells display local methylation disorder arising from epimutations that were discussed earlier (49, 57). These epimutational changes are reminiscent of the process of genetic diversification, resulting in cell-to-cell variability in local DNA methylation patterns. Furthermore, recent integrative epigenomic analysis incorporating histone modifications and DNA methylation demonstrated corrupted coherence across different strata of the CLL epigenome (69). As reported by Pastore et al, this manifests in specific chromatin regions simultaneously acquiring activating acetylation marks as well as repressing methylation marks, modifications that are normally mutually exclusive.

Local DNA methylation disorder, together with corrupted coordination of epigenetic modifications, thus generate enormous intraclonal epigenetic diversity in CLL. Such epigenetic heterogeneity has several consequences. First, the resultant assortment of different chromatin states within individual genomic loci leads to decreased epigenetic-transcriptional coordination, thereby introducing greater cell-to-cell discordance in gene expression (49, 69). This gives rise to enhanced CLL intraclonal transcriptional heterogeneity. Second, increased transcriptional heterogeneity inevitably translates into greater phenotypic variability. This results in a permissive CLL cell state that confers a higher level of plasticity wherein an admixture of cells with different epigenetic identities lowers the barrier for transition between cell states (3, 69). Third, a more permissive epigenetic landscape could also promote genetic clonal evolution by supporting the propagation of newly acquired genetic alterations to progeny CLL cells (49). Thus, epigenetic heterogeneity underpins intratumoral heterogeneity at multiple levels, together fueling CLL clonal evolution. Although unproven, one can reason that the level of epigenetic heterogeneity would increase in tandem with genetic evolution and clinical progression. Moreover, enhanced cell state plasticity that could corrupt differentiation programs and undermine differentiation hierarchies may hold particular relevance for Richter transformation.

### Microenvironmental Heterogeneity and Tumor-Immune Co-Evolution

Our understanding of clonal evolution in CLL is incomplete without considering the evolutionary dynamics of the myriad of interactions between CLL cells and their surrounding tumor immune microenvironment. The impact of the tumor microenvironment is evident from the tumor-supporting interactions upon which CLL cells depend for survival and proliferation. Notable examples of such interactions within lymph nodes include the CD40-CD40L interaction with follicular T cells (100), and the BAFF/APRIL-tumor necrosis factor receptor (TNF-R) interaction with nurse-like cells (101), as well as antigen-BCR interactions (102). On the other hand, the interactions that underpin anti-CLL immunity are less well characterized, but there is emerging *in vitro* and *in vivo* evidence for their existence (103–106). Capitalizing on advances in proteogenomic platforms and computational algorithms (62, 63), studies in recent years have begun to elucidate the repertoire of CLL tumor-associated antigens and neoantigens (103, 104, 107, 108). These studies revealed diverse neoantigen sources including somatic mutations (104), small insertions or deletions (indels) (107), splice variants and novel unannotated open-reading frames (nuORFs) (108), all capable of eliciting potent antitumor immune response. Recent work has also begun to unravel the immune cellular populations and tumor-immune interactions that may be important for antitumor immunity, notably within murine models of CLL (105, 106).

Cancer cells subvert antitumor immunity both by evolving strategies to evade immune detection and by suppressing the function of immune cells, leading to attenuated antitumor response. Tumor cells, for instance, can evade immune detection by downregulating tumor expression of MHC or otherwise interfering with the process of antigen processing or presentation, leading to loss of tumor antigen expression (10). These mechanisms have been suggested in CLL models (109), but have yet to be convincingly demonstrated in patients with CLL. However, CLL cells are known to upregulate immune inhibitory molecules such as PD-L1 (110). Moreover, CLL cells exert direct inhibitory effect on cytotoxic T cell function, resulting in impaired motility, immune synapse formation and cytotoxicity (111, 112). Chronic antigenic stimulation also renders T cells anergic, contributing to an exhaustion phenotype associated with functional impairment (113). In addition, natural killer (NK) cells, dendritic cells and monocytes are also functionally impaired in CLL, and the number of myeloid-derived suppressor cells (MDSCs), particularly of the polymorphonuclear MDSC subtype, are increased, which contribute to immune escape (114, 115). On the other hand, established and experimental CLL treatments such as ibrutinib, lenalidomide/avadomide, immune checkpoint inhibitors, and hematopoietic stem cell transplantation (HSCT) can reverse CLL-induced immune dysfunction (112, 116–122). Therefore, CLL-induced immune evasion and immunomodulatory treatments can be viewed as two opposing forces shaping the dynamic antitumor immune landscape.

The contribution of antitumor immunity to molding diverse CLL clinical trajectories is currently unclear. One can envision that whereas CLL progression coincide with immune escape, long-term disease stability as seen in patients with a highly indolent or spontaneously regressing clinical course, or in the majority of individuals with MBL who never progress to CLL, may be dependent to varying degree on immune control. This process is commonly known as cancer immunosurveillance (123). In relation to the latter, a recent study found differences in the inflammatory signatures between MBL and CLL (124). Likewise, sustained CLL remissions, particularly with novel therapeutic agents that modulate the immune system, may be contingent upon antitumor immunity, with immune escape presaging disease relapse. The evolving immune selection pressures and immunoediting processes, as well as the dynamics of immune cell states, tumor antigenic landscapes and tumor-immune interactions that govern CLL immune control and escape have not been fully characterized, particularly at the single-cell level, and represent important areas for future investigation.

## Patterns of Treatment-Induced Clonal Evolution in CLL

All too often, high-risk CLL progresses quickly to a clinical stage where treatment is required. With our expanding understanding of CLL pathobiology comes a paradigm shift in the therapeutic management of CLL, in which targeted therapies such as BCR signaling inhibitors (e.g. ibrutinib) and BCL-2 inhibitors (e.g. venetoclax) are increasingly replacing conventional chemotherapy-based treatments (125–127). Although the use of allogeneic HSCT (allo-HSCT) is also decreasing with the expanding availability of targeted agents, allo-HSCT remains an important therapeutic modality in multiply relapsed or refractory CLL (128–130). Moreover, immunotherapy is a burgeoning area of CLL research, as apparent from the flurry of recent research activity on chimeric antigen receptor (CAR) T and NK cells (131–133). Despite the transformative nature of novel therapies, therapeutic resistance inevitably arises. Of particular clinical relevance is how CLL cells evolve to become resistant to these new treatments. To illustrate the clonal evolutionary mechanisms that accompany resistance to targeted therapy and immunotherapy, we herein review recent work on ibrutinib, venetoclax and allo-HSCT.

### Convergent Evolution Leading to Resistance to CLL Targeted Therapy

The mechanism of resistance to targeted therapies frequently involves interfering with drug target binding or circumventing the target. The most comprehensively documented mechanism of acquired resistance to ibrutinib is mutations of the Bruton tyrosine kinase (*BTK*) or *PLCG2* genes (134, 135), reflecting ibrutinib as a BTK inhibitor that suppresses BCR signaling. *BTK* mutations confer ibrutinib resistance by prohibiting irreversible drug binding, with the *BTK-C481S* mutation as the most predominant. On the other hand, *PLCG2* gain-of-function mutations promote BTK-independent BCR signaling. With respect to venetoclax, *BCL2* mutations represent the most common resistance mechanism, with multiple studies reporting the detection of the *BCL2-G101V* mutation in association with clinical relapse (136–138). *BCL2* mutations result in diminished venetoclax binding to BCL-2, thus conferring drug resistance. Intriguingly, the *BTK*, *PLCG2* and *BCL2* mutations reported thus far have predominantly been subclonal (135, 136, 138, 139).

**Figure 4:**
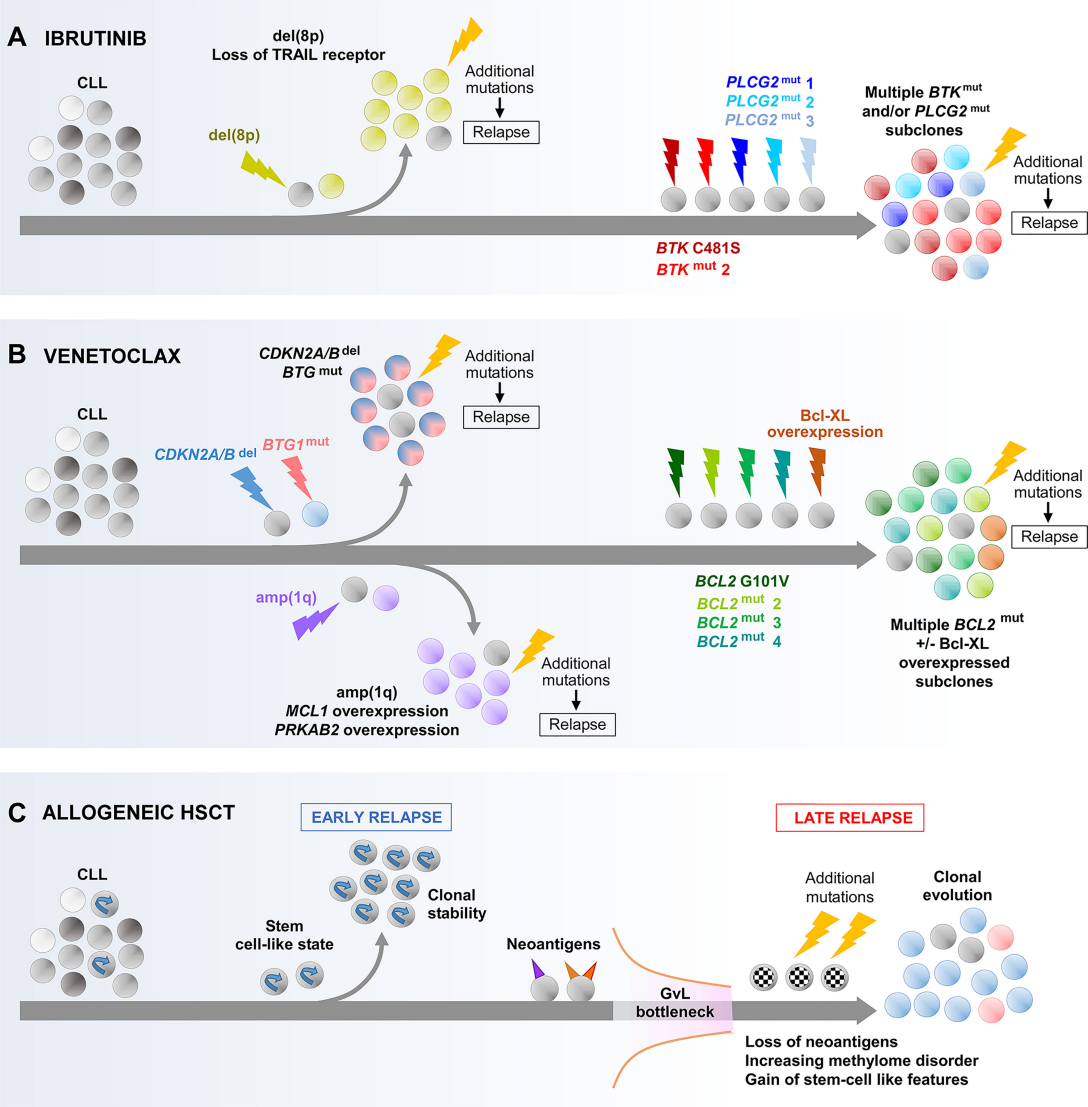
Main evolutionary paths towards the development of CLL resistance to ibrutinib **(A)**, venetoclax **(B)** and allogeneic HSCT **(C)**. As illustrated, multiple different routes can be undertaken by CLL that result in resistance to these treatments. In the case of resistance to ibrutinib or venetoclax through acquired *BTK*, *PLCG2* or *BCL2* mutations, the co-evolution of multiple subclones harboring different mutations of the same gene is shown which illustrates the concept of convergence evolution. *BTK*, Bruton tyrosine kinase; GvL, graft-versus-leukemia effect.

Longitudinal studies have provided insight into the evolutionary features associated with the acquisition of these mutations. While different evolutionary patterns have been observed, convergent evolution appears to be particularly common. For ibrutinib, this involves several different *BTK* or *PLCG2* mutations evolving in parallel (**Figure 4A**) (47, 139–141). For venetoclax, multiple *BCL2* mutations are likewise often seen to coexist (**Figure 4B**) (136–138). The CCFs of these variants within the same patient typically differ, suggesting that they arise within different subclones evolving independently, although definitive evidence will come from future single-cell analysis. These subclones may display different growth rates. For example, in the case of ibrutinib, Ahn et al. reported that *BTK*-mutated subclones often expand more rapidly than their *PLCG2-*mutated counterparts (139). Thus, one subclone may have growth advantage over other subclones, intrinsically or through the acquisition of additional alterations, and could achieve dominance over time.

Whether these mutations arise *de novo* during treatment or have pre-existed before treatment continues to be a matter of debate. Computational models support the existence of resistant subclones prior to treatment initiation (142), which has also been experimentally demonstrated in pre-treatment patient samples (141). Although in a proportion of patients the analysis of samples obtained near the start of treatment failed to detect resistance mutations that were identified in later samples (139), the detection of pre-existing mutations at low CCFs would likely have been limited by assay sensitivity. In any case, the simultaneous presence of multiple evolving subclones harboring alterations affecting identical genes demonstrates the enormous selection pressure that likely takes place during treatment, wherein CLL cells, through extensive trial and error, adapt by eventually evolving similar traits within distinct branches to create a cohesive resistant phenotype.

### Heterogeneous Evolutionary Paths to Ibrutinib and Venetoclax Resistance

Although the evolution of *BTK*/*PLCG2* or *BCL2* mutations represents a common mechanism of resistance to ibrutinib and venetoclax respectively, these mutations are not universally detected in therapy-resistant patients. Indeed, acquired *BTK* or *PLCG2* mutations are typically present in only 80% of individuals who relapse from ibrutinib (135). Likewise, acquired *BCL2* mutations were reported in only 7 of 15 patients (47%) relapsing from venetoclax in a recent study by Blombery and colleagues (136). Moreover, in some reported cases *BTK*/*PLCG2* or *BCL2* mutations remain at very low CCFs (<0.1) at the time of relapse (135, 136, 138, 139). This indicates that in some patients, other mechanisms are likely involved in mediating resistance to ibrutinib or venetoclax.

Two studies have analyzed WES data from longitudinal CLL samples to infer additional evolutionary routes to ibrutinib resistance (**Figure 4A**). In one study, Burger and colleagues identified the expansion of del(8p) subclones as a recurrent mechanism (141), occurring principally in patients without acquired *BTK* or *PLCG2* mutations within their CLL clones, which was corroborated by a separate study from Landau et al. (47). The deleted region in del(8p) involves the TRAIL receptor, a TNF-family extrinsic apoptotic receptor, the loss of which can be expected to enhance apoptotic resistance. Importantly, these studies showed that the fitness advantage conferred by del(8p) is conditional upon the acquisition of further genomic aberrations, without which the del(8p) subclone is not selected.

In the case of venetoclax resistance (**Figure 4B**), Blombery et al. showed that subclones with Bcl-xL overexpression and wild-type *BCL2* could coevolve alongside those harboring *BCL2* mutation (136). In addition, Herling and colleagues reported a recurring resistance mechanism in *BCL2* wild-type patients, characterized by the emergence and selection of CLL subclones harboring *CDKN2A/B* deletions and/or *BTG1* mutations (143), the former likely undermining cell cycle regulation, whereas the latter could contribute to apoptotic resistance and enhanced proliferation downstream of *BCL2* and *CDKN2A/B*. Similar to the evolution of del(8p) in ibrutinib-resistant cases, the presence of additional alterations appears to be a prerequisite for the selection of subclones harboring *CDKN2A/B* and *BTG1* defects with venetoclax. Finally, Guièze and colleagues reported amp(1q) as a recurrent lesion in a subset of venetoclax-resistant patients with wild-type *BCL2* (144). The amplified region contains *MCL1* and *PRKAB2*. As a gene encoding an anti-apoptotic protein with recognized roles in CLL, *MCL1* upregulation enhances apoptotic resistance (145). On the other hand, overexpression of *PRKAB2* was shown to confer metabolic advantage by increasing the capacity of CLL cells for oxidative phosphorylation, mediated through its regulatory role within the protein kinase A/AMP-activated protein kinase (PKA/AMPK) pathway (144).

As depicted by the examples of ibrutinib and venetoclax, there are multiple evolutionary paths of acquired resistance to targeted CLL therapies. Even accounting for the discovery of the aforementioned additional mechanisms, we would not have identified all the disparate genetic changes that CLL cells can accumulate on their road to therapeutic resistance. The heterogeneity of evolutionary mechanisms underpinning ibrutinib or venetoclax resistance, especially in patients without identifiable *BTK, PLCG2* or *BCL2* mutations, can potentially complicate further therapeutic targeting. On the other hand, the evolutionary dynamics at the transcriptional, epigenetic and phenotypic levels that accompany genetic evolution leading to ibrutinib or venetoclax resistance remain largely unexplored. In this respect, the work of Guièze et al. which applies genome-wide CRISPR screens and RNA-seq to cell line models has offered critical insight into important phenotypic mechanisms, for instance identifying metabolic dysregulation as a resistance mechanism to venetoclax (144). However, the application of integrative multidimensional single-cell analysis to longitudinal primary samples within uniformly treated patient cohorts, ideally within the setting of a clinical trial, will greatly enrich our understanding of the evolutionary processes underpinning resistance to targeted agents, potentially identifying common mechanisms and novel therapeutic targets.

### Tumor-Immune Dynamics in Response and Resistance to Targeted Therapy

In contrast to chemotherapy-based treatments, multiple studies have demonstrated the ability of ibrutinib to reverse T cell dysfunction in CLL (116–119). In particular, recent work by Baptista and colleagues showed that clinical response to ibrutinib is accompanied by the oligoclonal expansion of cytotoxic CD8^+^ T cells, which is reversed upon subsequent CLL progression (119). Moreover, these oligoclonal T cell populations were capable of eliciting potent anti-CLL cytotoxicity. Notwithstanding the impact of ibrutinib on CLL tumor burden that could confound their interpretation, these results suggest that tumor-immune co-evolutionary dynamics could potentially determine response and resistance to ibrutinib.

To assess the changes in CLL and immune cells that accompany response to ibrutinib, Rendeiro et al. recently carried out an integrative immunophenotypic, single-cell transcriptomic and chromatin mapping study on longitudinal peripheral blood samples from patients receiving ibrutinib therapy (146). Analysis of CLL cells revealed reduced NF-κB binding, curtailed activity of lineage-defining transcription factors, erosion of CLL cell identity, and the acquisition of quiescence-like transcriptome signature as features that characterize CLL response to ibrutinib. Peripheral blood T cells exhibited a quiescence-like gene signature, whereas monocytes and macrophages displayed an upregulation of inflammatory genes, both associated with defined chromatin accessibility changes. However, the assessment of tumor-immune dynamics in this study was hampered by a lack of lymph node and bone marrow samples. Moreover, the absence of CLL cases progressing on ibrutinib did not allow a comparison of response versus resistance to identify determinants of clinical outcome. Nevertheless, this study provides a relevant foundation upon which future studies can build. To comprehensively assess tumor-immune dynamics, longitudinal studies should ideally incorporate the analysis of tumor antigenic landscapes and tumor-immune interactions as well as immune cell states within the different microenvironmental compartments in which CLL cells reside.

In CLL, measurable residual disease (MRD) is predictive of long-term treatment outcome within various therapeutic settings (147–149), and is widely adopted as a surrogate endpoint as well as a guide to treatment duration within clinical trials. The study of tumor-immune co-evolution may be of particular relevance within MRD-adaptive treatment settings where a targeted therapy or therapeutic combination (e.g. ibrutinib plus venetoclax) is administered until MRD negativity is attained (150). It will be of importance, for instance, to ascertain whether differing tumor-immune dynamics impact upon MRD response kinetics. For example, more profound anti-CLL immune activity may characterize rapid responders and those individuals achieving sustained MRD negativity, compared to slow responders and others with short-lived treatment response. The findings of such an investigation could inform the potential use of immunomodulatory treatments to preempt CLL relapse.

### Clonal Evolution Underpinning CLL Relapse Following Allogeneic HSCT

Allogeneic HSCT exemplifies the power of harnessing antitumor immunity for cancer treatment, but relapses nevertheless occur. Recently, Bachireddy and colleagues investigated the evolutionary dynamics underlying resistance to graft-versus-leukemia (GvL) effect, uncovering mechanisms that may have potential broader relevance for CLL immunotherapy (151). Through longitudinal analysis integrating genetic, transcriptomic and epigenetic analyses, Bachireddy et al. identified two distinct evolutionary paths that give rise to early and late relapses respectively post allo-HSCT (**Figure 4C**). Early relapses are characterized by clonal equilibrium, and are underpinned by a pre-existing stem-like transcriptional state that confers resistance to GvL. In contrast, late relapses are characterized by clonal evolution secondary to a GvL selection pressure (‘GvL bottleneck’), wherein the more immunogenic subclones expressing neoantigens with stronger predicted MHC binding affinity, and hence likely to have attracted stronger GvL response, are selectively depleted. Late relapses are therefore mediated by CLL subclones with neoantigen loss and poor immunogenicity. Moreover, these subclones acquire a stem-like state through upregulating stem cell pathways. The latter is underscored by enhanced local DNA methylation disorder, particularly within the promoters of stem cell pathway genes. Thus, stem cell properties are important determinants of CLL relapse post allo-HSCT, with these properties having pre-existed within the CLL clone in instances of early relapse, and acquired in the case of late relapse.

As apparent from the above, the pattern of clonal evolution leading to relapse post allo-HSCT differs markedly from the evolutionary dynamics that mediate resistance to chemotherapy or targeted therapy. This serves to illustrate the vastly different selection pressures that are imposed by different treatment modalities. The work of Bachireddy et al. also exemplifies the use of integrative multidimensional approaches to dissect clonal evolution which allow us to appreciate the non-genetic and genetic determinants of clonal dynamics, a comprehensive understanding of which will enable us to devise better treatments to manage CLL relapse.

## Clonal Evolution Leading to Richter Transformation

**Figure 5:**
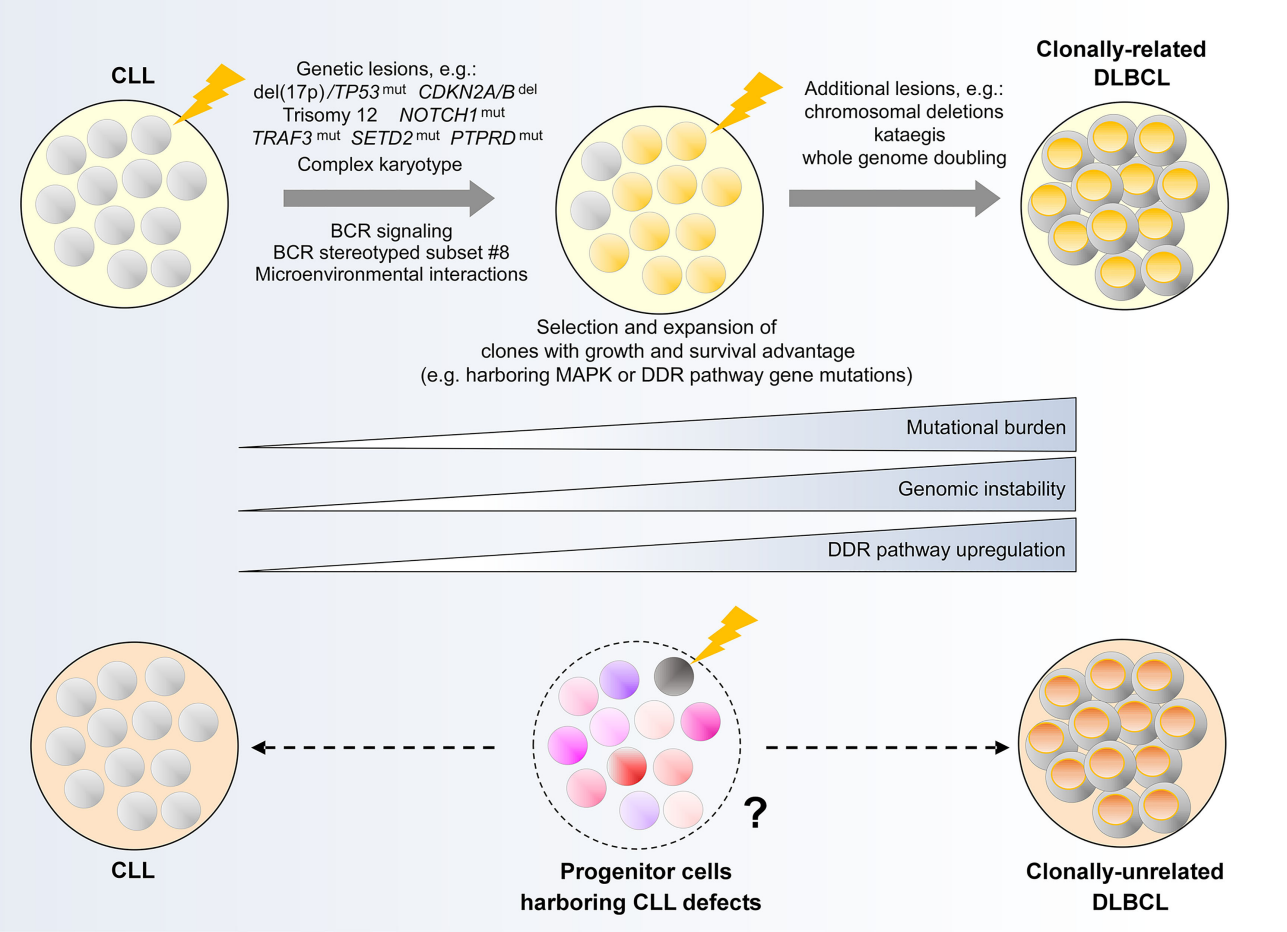
Clonal evolution underpinning Richter transformation of CLL to DLBCL. (Upper panel) Genetic alterations and biological processes implicated in the development of clonally-related Richter syndrome. (Lower panel) Hypothetical model illustrating a possible evolutionary path underlying clonally-unrelated Richter syndrome. MAPK, mitogen activated protein kinase; DDR, DNA damage response.

Richter Syndrome (RS), characterized by the high-grade transformation of CLL to diffuse large B cell lymphoma (DLBCL) and less commonly to Hodgkin lymphoma, represents a catastrophic clinical sequel of high-risk CLL. RS occurs in an estimated 2% to 10% of CLL patients (5). Individuals with RS respond poorly to currently available treatments, with dismal overall survival typically in the range of 3 to 6 months (152, 153). Richter transformation therefore represents the greatest current unmet need in CLL. There is compelling rationale to understand clonal evolution within this context, as an essential foundation for the development of improved therapeutics. On the basis of the clonal relationship between the antecedent CLL and the transformed lymphoma, RS can be classified as either clonally-related or unrelated, with identical or distinct IGHV rearrangements respectively (5). In the final part of our review, we will discuss our current understanding of the evolutionary processes underpinning these two types of RS (**Figure 5**), highlighting important areas for future investigation.

### Clonally-Related Richter Transformation

Two earlier studies have identified common genetic evolutionary routes which CLL cells undertake during the course of clonally-related Richter transformation. In the first study, Chigrinova et al. analyzed CLL and DLBCL samples from a series of patients who have undergone transformation to RS, identifying genetic lesions that are likely acquired prior to and during transformation using SNP array and targeted gene sequencing (154). This study revealed two genetic pathways leading to RS, the first involving *TP53* inactivation and/or *CDKN2A/B* loss, alongside *MYC* activation, and the second involving trisomy 12. In addition, *NOTCH1* mutations were found frequently in patients with RS, particularly among individuals also harboring trisomy 12. The second study by Fabbri et al. (155), which employed WES to interrogate CLL-RS pairs and additional Richter-transformed lymphomas, confirmed the prevalence of genetic lesions identified in Chigrinova et al. Moreover, this study revealed a predominantly linear evolutionary trajectory that accompanies Richter transformation, wherein the majority of CLL phase genetic alterations are maintained in the Richter-transformed lymphoma, with the acquisition of additional heterogeneous lesions at the time of transformation.

The role of the most commonly encountered genetic alterations in Richter transformation was explored in recent studies utilizing mouse models of CLL. Chakraborty and colleagues showed that the concurrent loss of *TP53* and *CDKN2A/B* led to an abolition of cell cycle control, allowing unrestrained BCR signaling-driven clonal proliferation that obviates the need for co-stimulatory signals (156). This is evidenced by the induction of a Richter transformation-like process within the Eµ-TCL1 model upon combined deletion of these two genes. In a separate study, Kohlhaas et al. showed that constitutive activation of Notch1 within Eµ-TCL1 mice recapitulated the Richter phenotype (157), thus substantiating the role of gain-of-function *NOTCH1* mutations in RS. Alternatively, at least within the Eµ-TCL1 model, it appears that constitutive activation of AKT, a component of the BCR signaling pathway, could drive Richter transformation through Notch1 activation in the absence of *NOTCH1* mutations. The latter seems to be mediated through an AKT-induced expansion of CD4^+^ T cells within the tumor microenvironment that express the Notch1 ligand DLL1 (157), indicating that genetic and non-genetic evolutionary mechanisms could converge on the same phenotypic outcome. Indeed, certain stereotyped BCR subsets, particularly subset #8, have been shown to confer increased risk of RS (20, 158). These findings highlight the need for multimodal clonal evolution studies on patients with RS that consider genetic and non-genetic mechanisms in equal measures.

In this context, Klintman and colleagues recently performed an integrative analysis of CLL-RS pairs in patients with RS that included transcriptomic analysis in addition to WGS, uncovering important biological processes during the evolution of CLL to high-grade lymphoma (48). First, RS is accompanied by an increase in mutational burden affecting large numbers of genes not previously implicated in CLL. These include recurrent mutations in *TRAF3*, *SETD2* and *PTPRD*, as well as genes with important roles in DDR or MAPK-RAS-ERK signaling. Preferential selection of subclones harboring these alterations was consistently observed. Second, in association with genetic evolution, transcriptome analysis revealed differential regulation of DDR genes. Coupled with prominent DDR-related mutational signatures, Klintman et al. highlighted the contribution of a corrupted DDR to genomic instability in RS, with genomic instability providing a permissive condition for the acquisition of further genomic events in the evolutionary process. These include CNAs and whole genome doubling, described recently by Parry et al. (159), as well as kataegis, reported by Klintman et al. (48). Recent work also demonstrates complex karyotype as a risk factor for the development of RS (31), with complex karyotype representing another consequence of genomic instability.

Taken together, these reports underscore multiple tumor-intrinsic and extrinsic factors that likely influence clonal dynamics in clonally-related RS. Further work should harness integrative single-cell technology to interrogate clonal dynamics and to resolve the interaction among genomic, transcriptomic, epigenomic and microenvironmental determinants of clonally-related RS. Further work should also apply the same technology to study clonal evolution in Richter transformation occurring during treatment with targeted agents such as ibrutinib or venetoclax. Studies thus far revealed diverse genetic features in these patients, with *BTK, PLCG2* or *BCL2* mutations being absent in many instances (96, 141, 143, 160, 161). Integrative analysis within larger cohorts will shed light on potential shared mechanisms in these individuals.

### Clonally-Unrelated Richter Transformation

In contrast to clonally-related Richter transformation, there has been minimal substantive work on the evolution of clonally-unrelated Richter transformation. A previous study by Lucas and colleagues showed that crossing Eµ-TCL1 mice with Eµ-Myc mice generated coexisting CLL and clonally-unrelated Richter-like lymphoma (162), suggesting that clonally-unrelated RS could potentially be MYC-driven. In addition, it would not be unreasonable to speculate that clonally-unrelated RS could originate from the evolution and subsequent transformation of HSCs or progenitor cells that harbor CLL/lymphoma genomic alterations (‘CLL/lymphoma reservoirs’), rather than from the transformation of the established CLL clone. This remains unproven and further work on longitudinal patient samples and animal models will undoubtedly enlighten our understanding of clonal evolution in this uncharted area.

## Conclusions and Perspectives

In this review, we provided a contemporaneous account of clonal evolution as it relates to high-risk CLL, highlighting recent discoveries that have offered novel insight. The past several years have seen profound shifts in our understanding of clonal evolution underpinned by a maturing definition of high-risk CLL and an increasing sophistication of next-generation sequencing technology. We have begun to understand the non-genetic sources of clonal heterogeneity, and the relevance of tumor-immune dynamics. We have also come to appreciate that with each therapeutic innovation comes the inevitable problem of therapeutic resistance, which can only be tackled through an exhaustive understanding of clonal evolution.

Amidst the seemingly diverse CLL evolutionary landscape, we have begun to identify some recurring patterns and commonalities; the convergent evolutionary patterns mediating resistance to both ibrutinib and venetoclax is a case in point. At the same time we recognize that many aspects of CLL clonal evolution remain unresolved. Why do some MBLs and CLLs progress, while others harboring similar genetic abnormalities remain stable for decades, or even spontaneously regress? In patients developing resistance to treatment, how do genetic, transcriptional, epigenetic, tumor antigenic and immune microenvironmental alterations converge to produce a shared resistant phenotype? Furthermore, what evolutionary mechanisms underpin Richter transformation, particularly in clonally-unrelated cases? The application of integrative single-cell technology within well-characterized patient cohorts and relevant disease models will spearhead advances in these areas and address these fundamental questions in the years to come.

An equally important consideration is how we can utilize and translate this newfound knowledge of CLL clonal evolution for the betterment of our patients. Already we know that intratumoral genetic heterogeneity and clonal evolution predict for shortened time to CLL progression (34, 44). On the other hand, how heterogeneity at the transcriptional, epigenetic and microenvironmental levels interacts with genetic heterogeneity in influencing CLL prognostication remains to be ascertained. Current prognostic models are constructed based on data derived from a single timepoint, for instance at diagnosis or prior to treatment, which provide only a limited snapshot of the individual CLL within its longitudinal evolutionary history. Intratumoral heterogeneity, subclonal architecture, growth dynamics and evolutionary trajectories, in contrast, are arguably more tangible measures of past behavior and potentially more reliable predictors of future outcome. These parameters may therefore have a place within future prognostic models so long as they can be easily assessed and quantified. Future translational efforts should therefore be directed at converting highly granular, genome-scale assessments of clonal evolution, which are laborious, expensive and generate enormous quantities of data, into assays that are equally informative but are also adequately scalable, reproducible and quantifiable to be used in the diagnostic setting and longitudinally for routine disease monitoring.

Routine monitoring of clonal evolution, in turn, may potentially open up a future ‘brave new world’ of personalized CLL medicine in which treatments are adapted according to subclonal dynamics and initiated preemptively to target subclonal outgrowth. Indeed, rising mutant CCFs of *BTK*, *PLCG2* or *BCL2* frequently anticipates disease progression and may signal the need for preemptive salvage treatment (135, 136, 139). The argument of whether therapeutic targeting should focus on clonal (truncal) or subclonal (branch) alterations has been extensively addressed within previous reviews (97, 163), and may be reconciled in the ideal scenario by the simultaneous targeting of truncal and branch lesions through rational treatment combinations. Targeting truncal lesions could eliminate the majority of the tumor load, while targeting branch lesions that confer the greatest survival and growth advantage may potentially avert selection of the most aggressive subclones. Our increasing knowledge of the non-genetic as well as genetic determinants of intratumoral heterogeneity lends itself to the future expansion of our therapeutic armamentarium to include novel treatments that target cellular dependencies unique to specific CLL subclones. These dependencies may arise from distinct tumor-immune interactions or from specific genetic, epigenetic or transcriptional alterations. Indeed, as alluded to in our review, immunotherapy is a rapidly developing area in CLL, and experimental investigations of epigenetic therapies, such as BET bromodomain inhibitors (164), are also emerging. It is therefore possible to envision a future in which the timing and choice of CLL treatment are guided by longitudinal monitoring of subclonal dynamics.

Finally, the concepts of evolutionary herding and clonal homogenization are gaining traction and may become feasible in the future world of evolution-adapted treatments. These proactive therapeutic strategies aim to maintain clonal equilibrium and reduce subclonal diversity, thereby impeding CLL progression and preventing relapse. Targeting concurrently trunk and branch lesions represents one way of achieving this, as is the simultaneous targeting of trunk lesions and any anticipated escape mechanisms or backup pathways pertaining to the truncal target. Evolutionary herding and clonal homogenization could also be achieved by therapeutically inhibiting the genetic or epigenetic mechanisms underpinning subclonal diversification, or by targeting subclones with the highest level of epigenetic plasticity or genomic instability which are most likely to further diversify and evolve. In relation to the latter, synthetically lethal strategies that target cellular dependencies specific to the most genetically unstable CLL subclones are being investigated (165); e.g. ATR pathway targeting of *TP53*-mutant subclones (166). Single-cell technology such as ClonMapper, which facilitates subclonal tracking and integrative analysis within *in vitro* and *in vivo* CLL models (50), will be hugely invaluable to this endeavor.

In conclusion, we have made massive strides in advancing our understanding of CLL clonal evolution over the past decade. Further research effort harnessing technological innovations will undoubtedly address current knowledge gaps and unanswered questions. Moreover, clinical translation of these advances has enormous potential to revolutionize prognostication and treatment of patients with CLL, bringing us closer to the ‘brave new world’ of tomorrow.

## Author contributions

MK and CW conceptualized the manuscript. MK drafted the manuscript. MK and CW edited and finalized the manuscript. All authors contributed to the article and approved the submitted version.

## Funding

MK is supported by a UK National Institute for Health Research (NIHR) clinical lectureship. This work is supported by grants from the National Institutes of Health (NCI-1RO1CA155010), the NIH/National Cancer Institute (NIH/NCI) (P01 CA206978, UG1CA233338) and the CLL Global Research Foundation.

## Conflict of Interest

CW holds equity in BioNTech, Inc. and receives research funding support from Pharmacyclics.

The remaining author declares that the research was conducted in the absence of any commercial or financial relationships that could be construed as a potential conflict of interest.

## Publisher’s Note

All claims expressed in this article are solely those of the authors and do not necessarily represent those of their affiliated organizations, or those of the publisher, the editors and the reviewers. Any product that may be evaluated in this article, or claim that may be made by its manufacturer, is not guaranteed or endorsed by the publisher.
